# Synergistic Genotoxic Effects of Gamma Rays and UVB Radiation on Human Blood

**DOI:** 10.3390/antiox14121451

**Published:** 2025-12-02

**Authors:** Angeliki Gkikoudi, Athanasia Adamopoulou, Despoina Diamadaki, Panagiotis Matsades, Ioannis Tzakakos, Sotiria Triantopoulou, Spyridon N. Vasilopoulos, Gina Manda, Georgia I. Terzoudi, Alexandros G. Georgakilas

**Affiliations:** 1 DNA Damage Laboratory, Physics Department, School of Applied Mathematical and Physical Sciences, National Technical University of Athens (NTUA), Zografou Campus, 15780 Athens, Greece; angelikigkikoudi@mail.ntua.gr (A.G.); ge19143@ntua.gr (A.A.); ge20004@mail.ntua.gr (D.D.); ge19811@mail.ntua.gr (P.M.); ge20031@mail.ntua.gr (I.T.); svasilopoulos@mail.ntua.gr (S.N.V.); 2 Health Physics, Radiobiology & Cytogenetics Laboratory, Institute of Nuclear & Radiological Sciences & Technology, Energy & Safety, National Centre for Scientific Research “Demokritos”, 15341 Agia Paraskevi, Greece; iro@rrp.demokritos.gr (S.T.); gterzoudi@rrp.demokritos.gr (G.I.T.); 3 Radiobiology Laboratory, “Victor Babeș” National Institute of Pathology, 99-101 Splaiul Independentei, 050096 Bucharest, Romania; gina.manda@ivb.ro

**Keywords:** gamma radiation, UVB radiation, genotoxic synergy, radiation biodosimetry, chromosomal aberrations, repair index, oxidative stress, redox imbalance, antioxidant defense

## Abstract

Exposure to ionizing and non-ionizing radiation from environmental and clinical settings can significantly threaten genomic stability, especially when combined. This ex vivo study investigates the potential combined effects of gamma radiation and ultraviolet B (UVB) exposure on human peripheral blood mononuclear cells (PBMCs) from healthy donors by exposing whole blood and isolated PBMCs to 1 Gy of gamma rays, to an absolute dose of approximately 100 J/m^2^ of UVB, or to their combination. Combined exposure resulted in significantly elevated γH2AX foci formation and chromosomal aberrations relative to individual stressors, with the most pronounced effects observed in isolated PBMCs. Notably, lymphocytes from some donors failed to proliferate after UVB or co-exposure. Based on our results, a predictive biophysical model derived from dicentric yield was developed to estimate the gamma-ray equivalent dose from co-exposure, indicating up to ~9% increase in lifetime cancer risk. Although this proof-of-concept study included only a small number of donors and focused on two endpoints (γH2AX and dicentric assays), it provides a controlled framework for investigating mechanisms of radiation-induced genomic instability. The results emphasize the importance of accounting for mixed radiation exposures in genotoxic risk assessment and radiation protection.

## 1. Introduction

Genomic integrity is compromised by a plethora of DNA-damaging agents coming from the environmental and clinical settings, especially when cells are exposed to multiple genotoxic stresses in sequence or combination. Two such agents extensively studied and documented for their ability to induce distinct but potentially complementary forms of DNA damage are ultraviolet B (UVB) and gamma radiation. UVB radiation (280–320 nm) primarily generates bulky DNA lesions such as cyclobutane pyrimidine dimers (CPDs) and 6-4 photoproducts, which can stall replication forks and activate error-prone repair while in parallel can cause replication stress and perturb cell cycle progression, further challenging the cell’s ability to maintain the mechanisms underlying DNA integrity and fidelity [1,2]. Gamma rays are high-energy photons that directly and indirectly cause DNA double-strand breaks (DSBs) and oxidatively clustered DNA lesions (OCDLs) [3]. Both ionizing and non-ionizing radiations are potent inducers of reactive oxygen and nitrogen species (ROS/RNS), which play dual roles as signaling molecules and as mediators of oxidative damage. The oxidative burst following radiation exposure not only drives direct DNA oxidation but also activates redox-sensitive transcription factors such as NF-κB and Nrf2, leading to inflammatory and adaptive antioxidant responses [4]. A number of in vivo and ex vivo studies have demonstrated that radiation-induced DNA damage is closely paralleled by characteristic oxidative biomarker responses, including antioxidant enzyme modulation and persistent oxidative DNA lesions (see Table S1 of Supplementary Section S1 for a structured overview of these benchmark studies). Among these, a range of chromosomal aberrations, including dicentric chromosomes and translocations, through misrepaired DSBs are also induced by gamma rays. These aberrations are critical biomarkers of genotoxicity and are tightly associated with carcinogenic potential and cell death [5,6]. Although the biological effects of UVB and gamma radiation have each been extensively characterized, relatively few studies have examined their combined action. Based on the limited existing reports, combined exposure to UVB and gamma rays results in an exacerbated burden of DSBs and increased risk of genomic instability, and this combined effect is particularly important in peripheral blood mononuclear cells (PBMCs), as they are sensitive to DNA damage and play a key role in immune function [7]. PBMCs were chosen because they serve as a well-established surrogate for human tissues—being easily accessible from blood samples and broadly reflective of human genetic, physiological, and immunological responses. To address these gaps, we investigated combined UVB and gamma-ray exposure in both PBMCs and whole blood to determine whether interaction effects occur that are missed in single-radiation studies and to clarify how the blood microenvironment influences DNA damage outcomes. Particularly, we aimed to explore the combined genotoxic effects of gamma radiation and UVB by examining dicentric chromosomes and γH2AX, as these are two well-known markers of DNA DSBs and their repair. Our findings will help us better understand how different genotoxic mechanisms work together and what this means for human health regarding environmental and therapeutic radiation exposure. Furthermore, using our data and other existing data, we created a predictive biophysical model based on dicentric chromosome analysis. This model offers a valuable tool for assessing genotoxic risk in scenarios involving complex radiation exposure and may aid in refining biodosimetric assessments in clinical or environmental contexts.

## 2. Materials and Methods

### 2.1. Donor Recruitment and Blood Collection

Blood samples (6–7 mL) from 7 healthy donors were collected in heparin-containing vials for either in vitro irradiation and cell culture initiation or for lymphocyte isolation, irradiation, and fixation, depending on the assay performed. Specifically, samples from three donors were used for the γH2AX immunofluorescence assay and samples from four donors for the chromosomal aberration assay. After the in vitro irradiation, the experimental procedures were performed according to the detailed protocols described in the next paragraphs. Written informed consent was obtained, as the project involves the use of human genetic material and biological samples, according to ethics protocol number 21/12/2023-17 (Date: 21 February 2023) NCSR ‘Demokritos’ bioethics committee. For additional information, see Section S2 in the Supplementary File. Ethical and logistical constraints associated with repeated blood collection and ex vivo irradiation experiments are the reasons of limited sample size. At this point we need to highlight that this study was designed to assess methodological feasibility and biological trends of mixed-field exposures rather than to draw population-level inferences.

### 2.2. Preparation of Samples and Irradiation Protocol

Peripheral blood (1 mL) was diluted 1:1 with unsupplemented RPMI 1640 medium and placed in 3.5 cm Petri dishes for irradiation. The dilution step was used only to ensure uniform UV exposure across the surface and does not alter the cellular composition; therefore, these preparations are referred to as “whole blood” throughout the text. No fetal bovine serum or other supplements were added at this stage. After irradiation, samples were incubated at 37 °C in a humidified atmosphere of 5% CO_2_ and 95% air for 20 min and 24 h prior to analysis. This partial dilution may have reduced plasma antioxidant concentrations and has to be noted as a study limitation of this study. PBMCs were then isolated using an equal volume of Lymphosep Separating Media (L0560, Biowest, Nuaillé, France), following standard procedures [8]. When directly irradiating PBMCs, isolation was performed before irradiation. Whole blood and isolated lymphocyte cultures were irradiated using a gamma radiation source of Cobalt-60 (Co-60) located at the National Centre for Scientific Research “Demokritos” in Athens, Greece. The Co-60 source delivers a dose rate of 0.10 Gy/min, ensuring consistent and precise irradiation exposure of samples. After approximately 20 min, samples kept at room temperature (RT) were exposed to UVB by using the UV Bench Lamp Model XX-15M, with a power output of 15 watts and a peak emission at 302 nm. UVB irradiation was performed at an absolute dose of 100 J/m^2^. This dose corresponds to a biologically relevant sub-cytotoxic exposure that balances between effective DNA damage signaling and not excessive apoptosis. The lamp was positioned 30 cm above the sample plane, yielding an effective irradiance (Eeff) of 5.6 ± 0.3 W/m^2^ as measured with a calibrated UVB radiometer. The exposure duration was 12.9 s, corresponding to an incident effective dose of approximately 72 J/m^2^, which translates to an absolute exposure of ~100 J/m^2^. The exposure times and distances were carefully controlled to deliver the desired UVB dose, and all procedures were conducted under standardized laboratory conditions to ensure reproducibility (Figure S1). For additional information, see Section S3 in the Supplementary File (Tables S2 and S3).

### 2.3. Dicentric Chromosome Assay

For dicentric chromosome analysis, whole blood and isolated PBMCs were cultured in RPMI 1640 medium containing 10% fetal bovine serum, 1% glutamine, and antibiotics [penicillin: 10,000 U/mL; streptomycin: 10,000 μg/mL (Sigma-Aldrich, Darmstadt, Germany)]. Phytohaemagglutinin (PHA) was dissolved in PBS at a concentration of 0.24 mg/mL. Cultures were incubated at 37 °C in a humidified incubator in an atmosphere of 5% CO_2_ and 95% air for 50 h. Colcemid solution was added 3 h before cell harvest (all treatments were processed using identical colcemid exposure times to ensure comparability across conditions), and cells were collected by centrifugation, treated in 75 mM KCl for 10 min, fixed in methanol: glacial acetic acid 3:1 (*v*/*v*) and processed for cytogenetic analysis. Giemsa staining was achieved by immersing slides in 2% Giemsa solution for 10 min, then washing with distilled water and air-drying. Slides were covered with coverslips and analyzed using a microscope (Axioplan 2, Carl Zeiss Microscopy GmbH, Hamburg, Germany). The chromosome aberration analysis was greatly facilitated by the dedicated IKAROS software (version V5.0 SR-27) for the semi-automated image analysis (MetaSystems, Altlussheim, Germany). The number of metaphases analyzed for each experimental time point was 500.

### 2.4. γH2AX Foci Analysis for Estimation of the DSBs and Repair

H2AX phosphorylation detection was performed by using a modification of the protocol of Tremi et al. [9]. Briefly, cell suspensions (PBMCs) were centrifuged on microscope slides using a Cytospin ROTANTA 460/460R centrifuge (Hettich, Tuttlingen, Germany), fixed for 15 min at RT with 3% paraformaldehyde (F8775, Sigma-Aldrich, Darmstadt, Germany) and 2% sucrose (A2211, Applichem GmbH, Darmstadt, Germany) in phosphate-buffered saline (PBS, 18912-014, Gibco, Grand Island, NY, USA), and then triple-washed with PBS. Cells were thereafter permeabilized for 10 min with 0.5% Triton X-100 (X100, Merck, Saint Louis, MO, USA) in 100 mM Tris-HCL pH 7.4 (A4263, Applichem GmbH, Darmstadt, Germany) and 50 mM EDTA pH 8 (A4982, AppliChem GmbH, Darmstadt, Germany) in distilled water, washed again three times with PBS, and blocked overnight at 2–8 °C with 0.5% bovine serum albumin (BSA, A7906, Sigma-Aldrich, Darmstadt, Germany) and 0.2% gelatin from cold water fish (G7041, Sigma-Aldrich, Darmstadt, Germany) in PBS. Cells were incubated for 90 min at RT with an antibody against histone H2AX (phospho S139) (NB100-384, Novus Biologicals, Abingdon, UK) in 0.5% BSA and 0.2% gelatin in PBS, and then were washed three times with PBS. A 90 min incubation at RT with goat anti-rabbit IgG H&L cross-absorbed secondary antibody labeled with Rhodamine Red-X (R-6394, Thermo Fisher Scientific, Waltham, MA, USA) in 0.5% BSA and 0.2% gelatin in PBS followed. After 3x washing with PBS, DNA was counter-stained with ProLong Gold Antifade Reagent with 4′6-diamidino-2-phenylindole (DAPI) (8961, Cell Signaling Technology Inc, Danvers, MA, USA), and cells were shielded with 22 × 22 mm coverslips, avoiding air bubble trapping. Two independent experiments were conducted for each donor. Microscope slides were analyzed using a Zeiss Axioskop-2 fluorescence microscope with ISIS software (version V5.0 SR-27). At least 100 cells per donor were analyzed with the JCountPro and JQuantPlus (Version 1.54p) image processing software (courtesy of Dr. Pavel Lobachevsky group, Peter McCallum Institute, Australia) [10].

## 3. Results

### 3.1. DNA Damage Measurement

**Figure 1:**
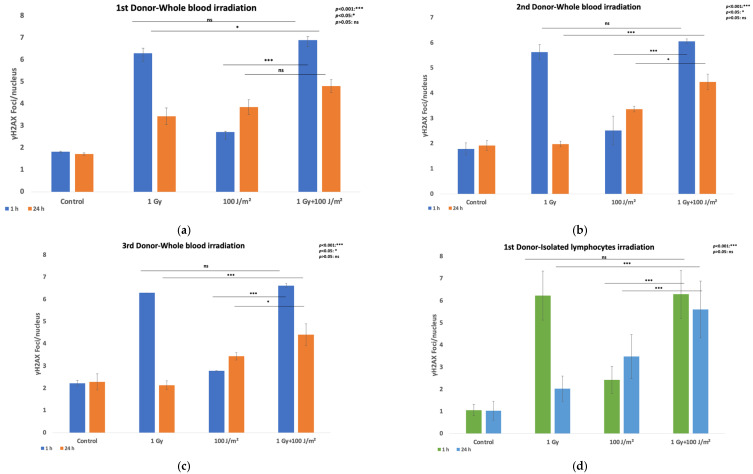

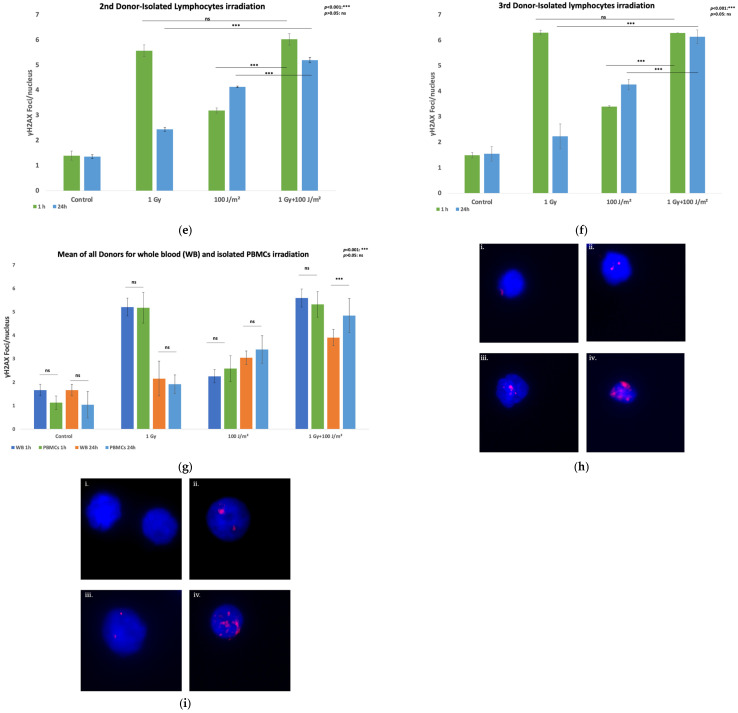
γH2AX staining in PBMCs following co-exposure to gamma rays and UVB. Results are presented as mean effect (value) ± standard deviation (SD) from 2 independent experiments. (**a**) γH2AX foci per nucleus in 1st donor PBMCs at 1 h and 24 h after exposure of whole blood to 1 Gy gamma rays or 100 J/m^2^ UVB as single stressors, or to combined challenges (gamma rays first, and UVB 20 min thereafter); (**b**) γH2AX foci per nucleus in 2nd donor PBMCs at 1 h and 24 h after exposure of whole blood to 1 Gy gamma rays or 100 J/m^2^ UVB as single stressors, or to combined challenges (gamma rays first, and UVB 20 min thereafter); (**c**) γH2AX foci per nucleus in 3rd donor PBMCs at 1 h and 24 h after exposure of whole blood to 1 Gy gamma rays or 100 J/m^2^ UVB as single stressors, or to combined challenges (gamma rays first, and UVB 20 min thereafter); (**d**) γH2AX foci per nucleus in 1st donor PBMCs at 1 h and 24 h after exposure of isolated PBMCs to 1 Gy gamma rays or 100 J/m^2^ UVB as single stressors, or to combined challenges (gamma rays first, and UVB 20 min thereafter); (**e**) γH2AX foci per nucleus in 2nd donor PBMCs at 1 h and 24 h after exposure of isolated PBMCs to 1 Gy gamma rays or 100 J/m^2^ UVB as single stressors, or to combined challenges (gamma rays first, and UVB 20 min thereafter); (**f**) γH2AX foci per nucleus in 3rd donor PBMCs at 1 h and 24 h after exposure of isolated PBMCs to 1 Gy gamma rays or 100 J/m^2^ UVB as single stressors, or to combined challenges (gamma rays first, and UVB 20 min thereafter); (**g**) Mean of all cultures for whole blood and isolated PBMCs irradiation; (**h**) Fluorescence images (γH2AX) of PBMCs following co-exposure of whole blood to gamma rays and UVB: (**i**) unirradiated, (**ii**) exposed to 100 J/m^2^ UVB, (**iii**) exposed to 1 Gy gamma rays and (**iv**) co-exposed to 1 Gy gamma rays and 100 J/m^2^ UVB all 24 h post-exposure (blue signal: cell nucleus, red signal: γH2AX foci); (**i**) Fluorescence images of PBMCs following co-exposure of isolated PBMCs to gamma rays and UVB: (**i**) unirradiated, (**ii**) exposed to 100 J/m^2^ UVB, (**iii**) exposed to 1 Gy gamma rays and (**iv**) co-exposed to 1 Gy gamma rays and 100 J/m^2^ UVB all 24 h post-exposure (blue signal: cell nucleus, red signal: γH2AX foci).

The evaluation of DNA damage responses in PBMCs following exposure to ionizing and non-ionizing radiation was performed by quantifying γH2AX foci per nucleus in both whole blood and isolated PBMCs from three different donors. Samples were exposed to 1 Gy gamma radiation or 100 J/m^2^ UVB, and to a combination of both stressors (gamma radiation followed by UVB 20 min later). Irradiated samples and sham-irradiated controls were analyzed at 1 h and 24 h post-exposure. Elevated levels of γH2AX foci were detected in whole blood samples (Figure 1a–c) at 1 h post-irradiation compared to controls, across all donors. The greatest increase was observed following 1 Gy exposure, with a moderate but consistent rise in foci after combined treatment. By 24 h, γH2AX foci generally declined toward baseline, although residual damage remained higher in the combined treatment group compared to the gamma rays exposed group, with a statistically significant difference in all donors. A similar trend was observed in isolated PBMCs (Figure 1d–f), with robust γH2AX induction at 1 h following gamma irradiation (1 Gy) and combined exposures to gamma rays and UVB. The combined treatment group consistently exhibited statistically significantly higher γH2AX foci number at the 24 h time point, suggesting additive or synergistic effects of sequential gamma and UVB exposure (for additional information on statistical analysis, please see Tables S4 and S5, Section S4 in the Supplementary File). Given the limited number of donors (n = 3), bootstrapping (1000 iterations) was applied to estimate 95% confidence intervals around mean foci and synergy metrics. The bootstrap results provided robust uncertainty estimates, while *t*-tests confirmed the significance and direction of the observed effect. Bootstrapped 95% confidence intervals confirmed consistent increases in γH2AX foci following exposure. In whole blood, γH2AX rose from 1.9 [1.8–2.1] (control) to 6.5 [6.2–6.8] (combined) at 1 h, with similar persistence at 24 h. PBMCs showed comparable trends. For additional information, see also Table S6 in Section S4 of the Supplementary File.

When averaged across all donors (Figure 1g), the data confirmed higher, but statistically significant only for the co-exposed condition in the 24 h time point, γH2AX foci induction in isolated PBMCs than in PBMCs irradiated in whole blood. Furthermore, while γH2AX foci levels declined by 24 h post-irradiation, they remained elevated relative to the baseline, especially in samples subjected to combined stressors. A representative dose–effect curve obtained from the first donor’s isolated PBMCs, illustrating the linear relationship between the absorbed gamma dose and γH2AX foci formation, is provided in Supplementary Figure S2.

Overall, results demonstrate an increased trend regarding the levels of DNA damage following combined exposure to gamma radiation and UVB, with whole blood PBMCs displaying a more pronounced response than isolated PBMCs. The time-dependent decrease in γH2AX foci suggests ongoing DNA repair, although residual damage remains evident at 24 h post-exposure. The increased γH2AX persistence at 24 h post co-exposure may indicate sustained oxidative stress, potentially exceeding the plasma antioxidant buffering capacity and leading to redox imbalance. This interpretation is consistent with patterns reported in previous studies that directly measured oxidative biomarkers alongside DNA damage endpoints (Supplementary Table S1), where persistent γH2AX foci were found to correlate with elevated 8-oxo-dG and antioxidant enzyme changes.

The assessment of DNA repair efficiency under all exposure conditions, both in whole blood and isolated PBMCs, was estimated with the calculation of the repair index (RI) for each donor (Table 1) by subtracting the number of γH2AX foci at 24 h (F_24_) from the number of γH2AX foci at 1 h (F_1_) and dividing to the number of γH2AX foci at 1 h, i.e., Repair index (RI) = (F_1_ − F_24_)/F_1_.

This metric provides a relative measure of DNA repair capacity. A lower repair index suggests limited repair or persistent damage (F_24_↑), while a higher repair index indicates a reduction in γH2AX foci, reflecting efficient DNA repair (F_24_↓). This calculation enables a direct comparison of repair dynamics between whole blood and isolated PBMCs. In whole blood samples, all three donors exhibited a decrease in γH2AX foci by 24 h as compared to the 1 h time point, with the repair index indicating more effective repair after 1 Gy exposure compared to combined treatment or UVB alone. The 3rd donor showed the lowest repair index for the co-exposure condition, suggesting slower repair or persistent damage, whereas the 2nd donor displayed a relatively better repair capacity overall. In isolated PBMCs, the repair index was generally lower than in whole blood in the co-exposure condition, indicating delayed or incomplete repair and greater susceptibility to persistent DNA damage. The attenuated genotoxic response in whole blood may suggest a protective role of endogenous antioxidants and redox-active plasma components, consistent with earlier observations of antioxidant quenching of UVB-induced ROS in skin [11].

### 3.2. Synergy Evaluation—The Bliss Independence Model

To better understand and evaluate the nature (additive or synergistic) of the combined action of gamma and UVB radiation and examine whether its induced biological effects are synergistic or just additive in terms of DNA damage, the Bliss Independence Model was used in both whole blood and isolated PBMCs, based on the detected foci counts. The Bliss Independence Model, built on the probability theory of independent events, is one of the most widely used synergy metrics. This model is mainly used to evaluate drug combination efficacy through the calculation of the expected combined effects of two drugs, and subsequent comparison with the corresponding observed effects [12,13].

All calculations and comparisons, the single activities of agents, as well as the observed effect of their combination, should be expressed as a probability between 0 and 1, meaning that all values used by the Bliss Independence Model should be normalized to a 0–1 scale before any calculation (https://gdsc-combinations.depmap.sanger.ac.uk/documentation, assessed 20 September 2025). Derived from the complete additivity of the probability theory, where the assumption that the agents act independently means that the combined effect is equal to the product of their individual effects, according to the formula: $\left(1-E_{AB\_exp}\right)=\left(1-E_{A}\right)\cdot \left(1-E_{B}\right).$ By solving this, we take the Bliss Independence Model formula:

(1) $$E_{AB\_exp}\;=E_{A}+E_{B}-E_{A}\cdot E_{B}$$

The method compares the observed effect ($E_{AB_{obs}}$) induced by the combination of agents A and B with the expected corresponding effect ($E_{AB\_exp}$), which was obtained based on the assumption that there is no agent-agent interaction effect. Typically, the combination effect is declared synergistic if $E_{AB\_obs}$ is higher than $E_{AB\_exp}\;$ [13]. For a more direct comparison, the Bliss excess is usually calculated as $\Delta =E_{AB\_obs}-E_{AB\_exp}$ and the effect induced by the combination of agents A and B is characterized according to the value of Δ, as summarized below:

(2) $$\Delta \left\{\begin{array}{c} >0,\;\;\;synergistic\;\\ =0,\;\;\;\;\;\;\;additive\;\\ <0,\;\;\;antagonistic\;\; \end{array}\right.$$

In this study, analysis was performed on γH2AX immunofluorescence data (foci counts) at 24 h post-irradiation in both isolated PBMCs and whole blood. We focused on the 24 h time point, as synergy—if present—is expected to emerge later rather than at 1 h. Experiments were conducted in three donors, with two replicates per donor (A and B). Mean values of foci counts were normalized to a 0–1 scale using the formula:

(3) $$E_{x}^{norm}=\;\frac{{\;\left(Foci\;count\right)}_{x}\;\;\;-\;control\;}{max\;observed\;\;-\;control}$$

here 0 = baseline (control) and 1 = maximal effect (highest foci count in co-exposure). In all cases, maximum damage occurred in the combined gamma and UVB exposure.

Under the Bliss model, gamma and UVB are considered independent, each inducing normalized effects E_γ_ and E_UVB_. The expected combined effect is:

(4) $$E_{gamma-UVB}^{exp}=\;E_{gamma}+\;E_{UVB}-\;E_{gamma}\cdot E_{UVB}$$

Having calculated the expected biological effect from the Bliss model (Table 2), to evaluate the combined action of gamma and UVB and characterize it as synergistic or just additive, Bliss excess values have been estimated based on the equation:

(5) $$\Delta =E_{gamma-UVB}^{obs}\;-\;E_{gamma-UVB}^{exp}$$

As shown from the table above (Table 2), for each donor and both in whole blood and in isolated PBMCs, all the values for the Bliss excess (Δ) were higher than zero. In accordance with the Bliss Independence Model, this indicates that gamma and UVB radiation, when combined, induce a biological effect that exceeds the effect of each radiation type alone, suggesting a potentially synergistic, rather than merely additive, interaction in terms of induced DNA damage. Observed, expected, and excess values for all donors and conditions are summarized in Table 2. In this case also, the 95% CIs for donor-mean Δ were estimated by bootstrapping. Analyses were performed in Python (v3.11). For additional information, see Table S6 in Section S4 of the Supplementary File.

To verify that the interaction of gamma and UVB is truly synergistic, we proceeded with a one-sample *t*-test. This approach allows for the comparison of the mean Bliss excess values ($\bar{\Delta }$) against zero, for investigating whether the observed synergy (in each donor in whole blood and in isolated PBMCs) was statistically significant. It should be noted that in the one-sample *t*-test the comparison of the mean Bliss excess was conducted against zero, since zero corresponds to the null hypothesis-namely, the absence of synergy.

The one-sample *t*-test revealed that the mean Bliss excess $\bar{\Delta }$ was significantly higher than zero in both experimental matrices (irradiated whole blood and isolated PBMCs), validating the synergistic action of gamma and UVB radiation in terms of induced DNA damage. More precisely, in whole blood, the mean $\bar{\Delta }$ value obtained from the three independent experiments was 0.32±0.15, with the *t*-test revealing that this mean was significantly higher than zero (*t*-test, t(2) = 3.63, *p* value = 0.034). Similarly, in isolated PBMCs the corresponding $\bar{\Delta }$, derived again from three independent experiments, was 0.30±0.09, with the *t*-test confirming a statistically significant difference from zero (*t*-test, t(2) = 5.96, *p* = 0.014), further supporting the presence of synergy in the combined action of gamma and UVB regarding radiation-induced DNA damage.

To test the robustness of the synergy analysis, we recalculated Bliss excess (Δ) using alternative normalization schemes, including global min–max, donor-wise min–max, and donor-wise z-score scaling. The results were consistent across all approaches, with Bliss Δ remaining positive in both whole blood and PBMCs (Table S7 in Section S4 of Supplementary File). To confirm that the observed synergy was not specific to the Bliss framework, we additionally evaluated the Highest Single Agent (HSA) model (Gaddum’s non-interaction criterion). HSA combination indices were <1 for all donors in both matrices, consistent with the Bliss-based results for synergy (see also Section S6 in Supplementary File). Statistical comparisons were performed using donor-level means (n = 3). Although the limited number of donors precludes formal population-level inference, the direction and magnitude of the effects were consistent across all individuals, and the bootstrapped confidence intervals were narrow, supporting the robustness of the observed synergistic trend.

### 3.3. Genomic Instability-Chromosomal Aberrations Assay

**Figure 2:**
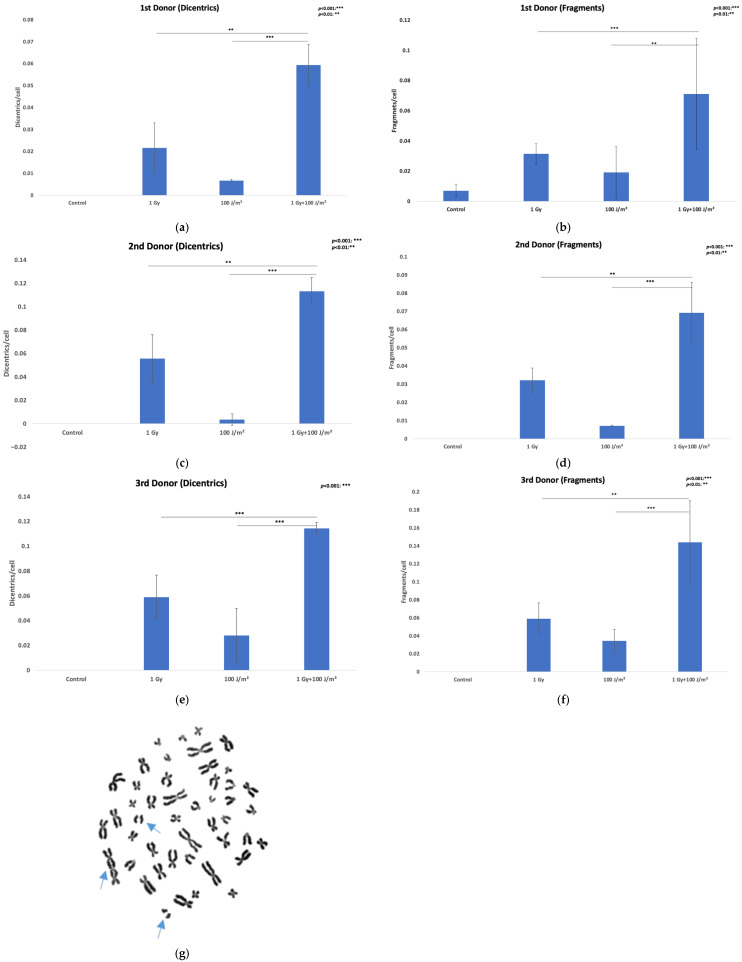
Chromosomal aberrations in lymphocytes. Results are presented as mean effect (value) ± SD for 2 independent experiments. (**a**) Dicentrics frequencies in PBMCs induced by exposure of whole blood to 1 Gy gamma rays or 100 J/m^2^ UVB as single stressors, or to combined challenges for the 1st donor; (**b**) Acentric fragments frequencies in PBMCs induced by exposure of whole blood to 1 Gy gamma rays or 100 J/m^2^ UVB as single stressors, or to combined challenges for the 1st donor; (**c**) Dicentrics frequencies in PBMCs induced by exposure of whole blood to 1 Gy gamma rays or 100 J/m^2^ UVB as single stressors, or to combined challenges for the 2nd donor; (**d**) Acentric fragments frequencies in PBMCs induced by exposure of whole blood to 1 Gy gamma rays or 100 J/m^2^ UVB as single stressors, or to combined challenges; (**e**) Dicentrics frequencies in PBMCs induced by exposure of whole blood to 1 Gy gamma rays or 100 J/m^2^ UVB as single stressors, or to combined challenges for the 3rd donor; (**f**) Acentric fragments frequencies in PBMCs induced by exposure of whole blood to 1 Gy gamma rays or 100 J/m^2^ UVB as single stressors, or to combined challenges for the 3rd donor; (**g**) Metaphase spread of PBMCs whole blood exposure to 1 Gy gamma rays and 100 J/m^2^ UVB with a tricentric chromosome and two acentric fragments (blue arrows).

Chromosomal instability was evaluated by quantifying dicentric chromosomes (dic) and acentric fragments (ace) in lymphocytes from three donors following exposure to 1 Gy gamma rays, 100 J/m^2^ UVB, or a combination of both (gamma rays followed by UVB 20 min later). The data are presented as mean aberrations per cell ± SD from two independent experiments. In all three investigated donors (Figure 2a–f), exposure to 1 Gy gamma rays induced a notable increase in both dicentrics and acentric fragments compared to control values. UVB exposure alone resulted in minimal chromosomal damage, whereas combined treatment (1 Gy gamma rays and 100 J/m^2^ UVB) consistently led to the highest levels of aberrations (Figure 2g). Dicentric frequencies were statistically significantly higher in the co-exposure condition compared to 1 Gy alone (χ^2^ test: χ^2^(1) = 9.68, *p* = 0.002 for 1st donor, χ^2^(1) = 10.27, *p* = 0.0013 for 2nd donor; and χ^2^(1) = 11.56, *p* = 0.0007 for 3rd donor). A substantial increase in acentric fragments was also observed (χ^2^ test: χ^2^(1) = 20.47, *p* = 1 × 10^−5^ for 1st donor; χ^2^(1) = 7.63, *p* = 0.006 for 2nd donor; and χ^2^(1) = 7.18, *p* = 0.007 for 3rd donor). When comparing co-exposure to UVB alone, dicentric frequencies were significantly higher for all donors (χ^2^ test: χ^2^(1) = 13.99, *p* = 0.0002 for 1st donor; χ^2^(1) = 30.93, *p* = 1 × 10^−6^ for 2nd donor; and χ^2^(1) = 32.92, *p* = 1 × 10^−7^ for 3rd donor). A significant increase in acentric fragments was again observed (χ^2^ test: χ^2^(1) = 10.55, *p* = 0.0012 for 1st donor; χ^2^(1) = 28.35, *p* = 2 × 10^−7^ for 2nd donor; and χ^2^(1) = 42.98, *p* = 1 × 10^−9^ for 3rd donor). Bootstrapped 95% confidence intervals confirmed consistent increases in dicentric frequency, which increased from ~0 (control) to 0.10 [0.07–0.12] dic/cell (combined). For additional information, see also Tables S6 and S7 in Section S4 of the Supplementary File.

**Figure 3:**
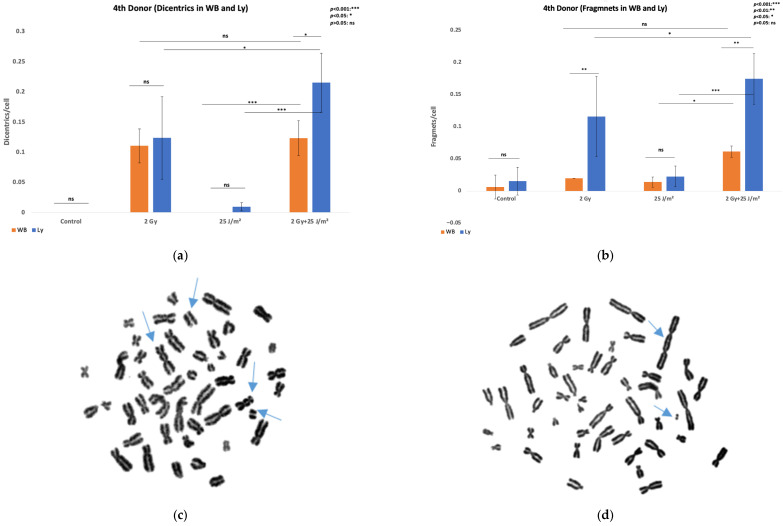
Chromosomal aberrations in lymphocytes of 4th donor. (**a**) Dicentrics frequencies in lymphocytes induced by exposure of whole blood and isolated PBMCs to 2 Gy gamma rays or 25 J/m^2^ UVB as single stressors, or to combined challenges; (**b**) Acentric fragments frequencies in PBMCs induced by exposure of whole blood and isolated lymphocytes to 2 Gy gamma rays or 25 J/m^2^ UVB as single stressors, or to combined challenges; (**c**) Metaphase spread of isolated lymphocytes exposed to 2 Gy gamma rays and 25 J/m^2^ UVB with two dicentric chromosomes and two acentric fragments (blue arrows).; (**d**) Metaphase spread of PBMCs whole blood exposure to 2 Gy gamma rays and 25 J/m^2^ UVB with a dicentric chromosome and an acentric fragment (blue arrows).

Chromosomal aberration analysis was also attempted in isolated lymphocytes from the third donor for the same exposure conditions. Isolated lymphocytes failed to yield analyzable metaphase spreads following UVB or combined treatment. The mitotic index (MI), expressed as mitoses per 1000 cells, decreased following gamma rays, UVB, and combined exposure compared to control (MI_control_ = 6.8× 10^−3^, MI_1Gy_ = 5.6 × 10^−3^, MI_100J/m2_ = 0 and MI_1Gy + 100J/m2_ = 0), indicating a cytostatic trend consistent with reduced proliferative activity. To investigate whether this proliferative inhibition could be overcome, additional experiments were performed using a higher gamma ray dose of 2 Gy and a lower UVB fluence of 25 J/m^2^ for a 4th donor. These irradiation conditions were applied to both whole blood and isolated lymphocytes. Results are shown in Figure 3 and further support the differential responses and DNA damage outcomes between whole blood and isolated lymphocyte preparations (χ^2^ tests). Comparison of co-exposed samples with those irradiated only with gamma rays in isolated lymphocytes showed significant increases under combined exposure for dicentrics (χ^2^(1) = 9.35, *p* = 0.002) and for acentric fragments (χ^2^(1) = 4.35, *p* = 0.037). In contrast, for whole-blood lymphocytes, dicentrics (χ^2^(1) = 0.095, *p* = 0.76) and fragments (χ^2^(1) = 2.27, *p* = 0.13) in co-exposed samples were not significantly higher compared to gamma-irradiated samples. Following the same pattern, comparison of co-exposed samples with those irradiated only with UVB in isolated lymphocytes revealed significant increases under combined exposure for dicentrics (χ^2^(1) = 93.22, *p* < 10^−21^) and for acentric fragments (χ^2^(1) = 56.88, *p* lh; 10^−13^). However, unlike the comparison with gamma rays, whole-blood lymphocytes also showed significantly higher yields under combined exposure compared with UVB alone for both dicentrics (χ^2^(1) = 18.48, *p* = 0.000017) and acentric fragments (χ^2^(1) = 4.15, *p* = 0.042). Comparison between PBMCs and whole blood lymphocytes for dicentric formation showed no significant differences in the control, 2 Gy, or 25 J/m^2^ groups (*p* > 0.05). In contrast, the combined exposure resulted in a significantly higher dicentric yield in PBMCs compared to whole blood (χ^2^(1) = 4.84, *p* = 0.028). For acentric fragments, no significant difference was observed between PBMCs and whole blood in the control and 25 J/m^2^ groups (*p* > 0.05). However, after 2 Gy exposure, fragment frequencies were significantly higher in PBMCs (χ^2^(1) = 8.22, *p* = 0.004), and this difference became even more pronounced under combined exposure, where PBMCs showed elevated fragment yield compared to whole blood (χ^2^(1) = 8.27, *p* = 0.003). Together, these results indicate that dicentric formation differs between PBMCs and whole blood only under combined radiation and UVB exposure, whereas acentric fragment formation is already elevated in PBMCs after 2 Gy irradiation alone and becomes strongly amplified by combined treatment.

### 3.4. Extension of the Linear-Quadratic Model to Predict Dicentric Chromosome Yield Following Co-Exposure of Whole Blood or Isolated PBMCs to Gamma and UVB Radiation

The elevated dicentric frequency cannot be explained by additive effects alone and aligns with observations of synergistic interactions between UVB and ionizing radiation exposures. To better capture this phenomenon, we extended the traditional linear-quadratic model by incorporating an exponential synergy term, as supported by our mechanistic and empirical findings. The standard LQ model, described by the expression Y = αD + βD^2^, where Y is the yield of dicentrics per cell and D is the dose of ionizing radiation, captures the linear contribution (α) of single radiation-induced events and the quadratic component (β) associated with interactions between two DNA damage events [14]. To accommodate non-ionizing UVB radiation, an additional linear term γD_UVB_ was introduced, where γ represents the independent contribution of UVB to dicentric formation—typically small or negligible under most exposure conditions.

Crucially, a synergy term δD_DUVB_·e^(−Δt/20)^ was included to model the interaction between gamma rays and UVB, where δ is the synergy coefficient, D and D_UVB_ are the respective doses, and Δt is the time interval between exposures, reflecting temporal effects on biological repair processes. This term describes the enhanced DNA damage observed when co-exposure overwhelms repair mechanisms, resulting in a non-linear increase in dicentrics. Importantly, w_order_, order index, is a dimensionless parameter that encodes the sequence of exposure: it equals 1 when gamma radiation follows UVB (a configuration known to produce stronger synergistic effects due to UVB-induced replication stress or repair interference) and is set to 0.1–0.2 when the order is reversed, while being set to 0.5 for simultaneous exposures [15]. This index ensures that the model reflects empirical observations where the timing of radiation delivery significantly influences dicentric yield. A residual error term ε is incorporated to account for biological variability and measurement uncertainty. The proposed extension of the linear-quadratic model is given by the equation:

Υ_Dic/cell_ = α·D + β·D^2^ + γ·D_UVB_ + δ·D·_UVB_·e^(−Δt/20)·^w_order_ + ε (6)

Using parameter values derived from the literature and our experimental setup (α = 0.05, β = 0.01, δ = 0.007, w_order_ = 0.1, Δt = 15, D = 1 Gy, D_UVB_ = 100 J/m^2^, ε = 0.01), the model predicted a dicentric yield of 0.103 dicentrics/cell under co-exposure conditions. Parameters were fitted to the combined dataset of the present study and literature values (see Supplementary Section S7).

Compared to our results, the model accurately captured the observed yields for donors 2 and 3, with deviations of less than 10%, confirming its validity in simulating co-exposure-induced genomic instability. For donor 1, the observed yield was substantially lower than predicted, suggesting individual variability in radiosensitivity or DNA repair capacity. These results underscore the predictive utility of the extended LQ model, particularly in mixed-radiation contexts, and highlight the importance of introducing exposure timing and sequence when modeling complex biological responses. To further evaluate the predictive accuracy of our extended linear-quadratic model, we applied it to WB data from the fourth donor exposed to 2 Gy gamma radiation or 25 J/m^2^ UVB, and their combination. The model predicted dicentric yields of 0.150, 0.010, and 0.167 dicentrics/cell for the respective conditions. Having obtained 0.11 for 2 Gy only, 0.01 for UVB only, and 0.12 for the combined exposure, the model performed well for UVB and reasonably well for gamma radiation, but in the co-exposure condition did slightly overpredict the dicentric yield by about 0.047 dicentrics/cell.

This probably outlines the biological “buffering” and protective capacity of whole blood and the potential moderating effect it has over the synergism in relation to isolated lymphocytes, as explained in greater detail in the following section. Despite this limitation, the model successfully captures both the direction and magnitude of the observed synergy, supporting its applicability in mixed-radiation biodosimetry and highlighting the importance of sample context (whole blood vs. isolated lymphocytes) in interpreting cytogenetic outcomes.

## 4. Discussion

While this study was limited to seven donors for ethical and logistical reasons, the reproducibility of treatment effects across individuals and the stability of bootstrapped confidence intervals suggest that the findings are not driven by inter-individual outliers. Instead, they reflect a consistent biological response to mixed-field exposures, underscoring the feasibility and interpretive value of this proof-of-concept design. The synergistic DNA damage observed is consistent with cumulative oxidative stress generated by both ionizing and non-ionizing radiations. Gamma radiation induces water radiolysis, producing hydroxyl radicals and hydrogen peroxide [16], whereas UVB generates superoxide and singlet oxygen at the cell surface [17]. Together, these processes increase the overall oxidative burden and redox imbalance. Significant differences were revealed through γH2AX foci analysis of DNA damage between isolated PBMCs and whole blood only following co-exposure condition (1 Gy gamma rays and 100 J/m^2^ UVB) 24 h post-irradiation (*t*-test, *p* < 0.001). The similar trend observed 1 h post-exposure in the response mechanism between whole blood and isolated PBMCs most likely reflects the direct induction of DSBs, which occurs before secondary protective mechanisms become relevant, as early γH2AX foci formation primarily reflects the physical induction of DNA DSBs, which occurs similarly in isolated PBMCs and whole blood.

**Figure 4:**
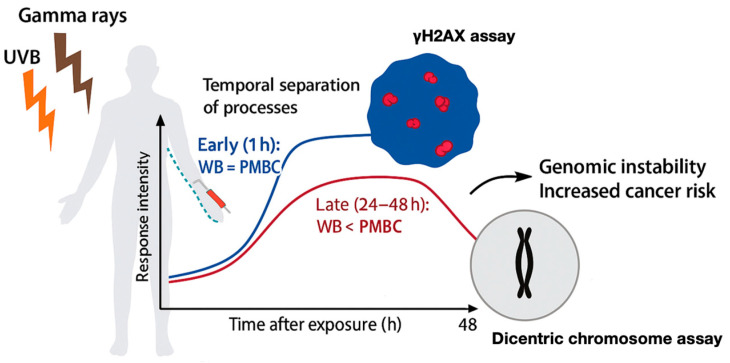
Schematic representation of biomarker-based assessment of genomic instability following gamma ray and UVB exposure. Peripheral blood mononuclear cells are analyzed using γH2AX immunofluorescence assay to quantify DNA DSBs and dicentric chromosome assay to measure chromosomal aberrations. Both endpoints contribute to the evaluation of genomic instability after co-exposure. The diagram summarizes the temporal separation of processes, emphasizing how similar early DNA damage can lead to distinct late outcomes depending on the cellular microenvironment. Created in Biorender. Gkikoudi. (2025) https://BioRender.com.

For the co-exposure condition a similar pattern emerged when chromosomal damage was evaluated via the dicentric assay. In the 1 Gy gamma rays and 100 J/m^2^ UVB co-exposure group, isolated lymphocytes exhibited significant cell death, precluding dicentric scoring, whereas lymphocytes in whole blood survived and displayed dicentric yields consistent with synergistic damage. It is known that dicentric formation requires cells to survive and progress through mitosis, processes that are influenced by the environment. Thus, while initial DSB induction appears equivalent, the expression of chromosomal damage diverges due to differences in post-irradiation survival and repair capacity. This temporal dissociation between early and late endpoints is schematically illustrated in Figure 4. While γH2AX foci reflects early and direct DSB formation [18], the dicentric assay is more sensitive to cumulative biological effects, including survival and repair dynamics and by using both γH2AX foci analysis and dicentric chromosome assay in mixed radiation exposures a comprehensive assessment of DNA damage and genomic instability it might been given. Together, these biomarkers form a synergistic paradigm for understanding radiation-induced genomic instability, capturing both acute DNA damage signaling and its downstream cytogenetic consequences. Crucially, their combined use emphasizes the need for multi-endpoint methods for precise biodosimetry and risk evaluation in situations where ionizing and non-ionizing radiation exposure occurs sequentially or simultaneously.

**Figure 5:**
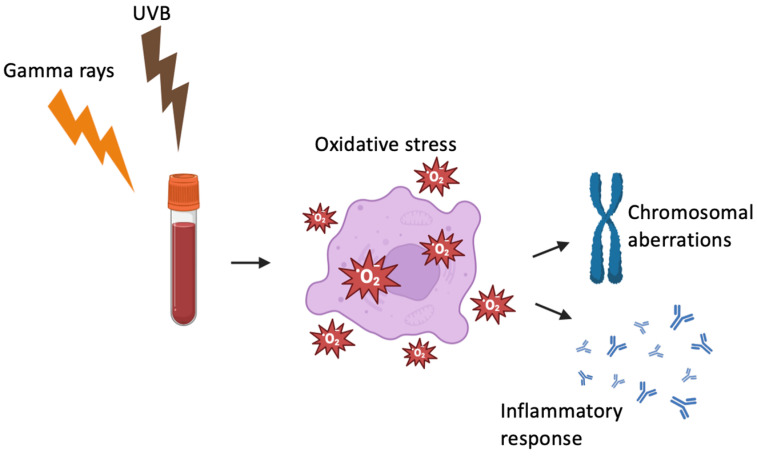
Radiation-induced oxidative stress leading to genomic instability and inflammation. Both gamma rays and UVB can trigger the generation of ROS in exposed biological samples. The resulting oxidative stress leads to cellular damage, including DNA and chromosomal aberrations, and activates redox-sensitive signaling pathways that promote inflammatory responses. Created in Biorender. Gkikoudi. (2025) https://BioRender.com.

These results imply that radiation responses are strongly influenced by the extracellular environment. Our findings align with growing evidence that blood, particularly its plasma components, plays a significant protective role against UVB-induced oxidative damage. Plasma contains a variety of endogenous antioxidants—including uric acid, vitamin C, and thiol-containing proteins—which scavenge reactive oxygen species (ROS) generated during UVB exposure [19]. Human serum albumin (HSA), via its Cys34 thiol, contributes roughly 40–70% to this capacity, and, in some contexts, the thiol itself accounts for up to 80% of albumin’s radical-scavenging activity [20,21,22,23]. Uric acid (urate) provides over half of the antioxidant potential in plasma [24], acting as a scavenger of ROS such as peroxyl, and hydroxyl radicals [25]. In addition, erythrocytes contain robust enzymatic antioxidants—catalase, glutathione peroxidase, and superoxide dismutase—that constitute a first-line defense against ROS in the blood, rapidly detoxifying hydrogen peroxide and superoxide anions [26,27]. This multienzyme system is essential for protecting hemoglobin and maintaining redox balance in circulating blood. By lowering extracellular ROS levels, erythrocytes likely mitigate ROS-mediated secondary oxidative DNA damage in co-circulating lymphocytes. In parallel, the presence of erythrocytes lowers local oxygen tension, since dissolved O_2_ is rapidly bound and consumed, and this reduces the oxygen enhancement effect (OER) of low-LET radiation [28,29]. Furthermore, platelet-rich plasma (PRP), a blood derivative enriched with growth factors and antioxidants, has been shown to reduce UVB-induced apoptosis, decrease pro-inflammatory cytokines, and enhance antioxidant enzyme activities such as superoxide dismutase (SOD) and glutathione peroxidase [30,31]. Collectively, these observations support the concept that blood is not merely a passive reservoir for oxidative stress biomarkers but an active biochemical and cellular shield mitigating UVB-induced damage (Figure 5). Given that PBMC isolation entails extended processing time, a reduction in endogenous antioxidant capacity during this interval cannot be excluded and may have contributed to the elevated DNA damage observed in isolated PBMCs relative to whole blood. A methodological limitation of this work is that whole blood was diluted 1:1 with unsupplemented RPMI prior to irradiation to ensure uniform UVB exposure across the sample surface. Although this dilution step does not alter cellular composition, it likely reduced endogenous plasma antioxidant concentrations. As a result, the magnitude of UVB and co-exposure induced DNA damage observed in the “whole blood” condition should be interpreted with caution, as it may not fully reflect the in vivo antioxidant buffering present in undiluted human blood.

Quantitative benchmarking against published human and animal studies in which γH2AX responses parallel oxidative biomarker changes indicates that the magnitude of the effects observed here corresponds to a moderate-to-high oxidative stress state. Specifically, a γH2AX increase in the size identified under co-exposure has been consistently associated with ~1.5–2-fold elevations in 8-oxo-dG levels, 10–25% increases in antioxidant enzyme activities (SOD, GPx, CAT), and 5–15% reductions in total antioxidant capacity (FRAP) in plasma and tissues. These estimates provide numerical context to the inferred redox imbalance even though biochemical antioxidant measurements were not performed directly in the present study (Table S1 is in the Supplementary File). We need to emphasize that this quantitative benchmarking is derived from published datasets and not from direct biochemical measurements in our samples and it is provided solely to contextualize the biological magnitude of the effects observed.

The estimated repair index further supports the differences in long-term cellular outcomes, as across all donors and exposure types, isolated PBMCs consistently showed lower values than whole blood, particularly in co-exposure conditions, indicating a reduced ability to resolve DNA damage over time among with lower proliferation capacity. Although the observed MI reduction supports a cytostatic effect, no direct viability or apoptosis markers (e.g., Annexin V/PI, caspase activation) were measured. Therefore, the interpretation of cytotoxicity is limited, and MI should be considered an indirect indicator of growth inhibition. The exposure to a sub-toxic dose of 2 Gy gamma rays and 25 J/m^2^ UVB showed a synergistic increase in dicentrics in the remaining viable isolated lymphocytes, with no effect in whole blood, suggesting that plasma-mediated protective mechanisms blunted the synergy. The observed discrepancy between isolated lymphocytes and whole blood may also reflect the intrinsic protective and reparative properties of the blood matrix.

A key observation from our study is the persistence of elevated γH2AX foci at 24 h after UVB exposure. While γH2AX is typically associated with the immediate marking of DSBs, its persistent presence long after irradiation might suggest incomplete resolution of DNA damage or ongoing secondary break formation. γH2AX persistence can also arise from replication stress, stalled replication forks, or persistent single-stranded regions that activate ATR-dependent signaling [32,33]. As previously reported by Freeman et al. [34], lesions in lymphocytes generated by UVB irradiation can persist due to the limited efficiency of nucleotide excision repair (NER) in non-dividing cells and therefore residual γH2AX at 24 h might reflect prolonged repair or replication stress signaling among with accumulation of unrepaired or misrepaired lesions and complex DNA damage formation. This broader interpretation aligns with the multifunctional role of γH2AX as a general sensor of DNA and chromatin stress.

Aside from qualitative observations of increased DNA damage during co-exposure, our statistical analysis using the Bliss Independence Model and Highest Single Agent Model provides quantitative evidence that gamma and UVB radiation interact synergistically at the cellular level rather than additively, as evidenced by the positive Bliss excess scores in all cases. These findings suggest that gamma and UVB radiation cause DNA damage via independent but complementary mechanisms, overwhelming repair pathways and amplifying genotoxic effects when stressors are combined leading to increased chromosomal instability.

One of the most serious and well-documented consequences of ionizing radiation exposure is an increased lifetime chance of cancer, which is thought to be the primary stochastic effect. According to the BEIR VII Phase 2 report [35], a dosage of 1 Gy from low-LET radiation, such as gamma rays, is related to an estimated 5% increase in lifetime risk for all solid malignancies in an adult population. This estimate is based on epidemiological studies of atomic bomb survivors, medical patients, and occupationally exposed populations, and it employs the linear no-threshold (LNT) model. In this study, we evaluated the cytogenetic impact of single and combined exposures to ionizing (gamma rays) and non-ionizing (UVB) radiation in peripheral blood lymphocytes using the γH2AX immunofluorescence and dicentric chromosome assay. Our results through chromosomal aberration analysis for the 2nd donor demonstrate that while 1 Gy of gamma rays alone produced a mean dicentric frequency of 0.055, the co-exposure condition resulted in a significantly higher frequency of 0.113 dicentrics per cell. This marked increase suggests a synergistic interaction between the two radiation types, leading to enhanced chromosomal instability.

To interpret the biological significance of this co-exposure, we estimated the equivalent gamma ray dose that would be required to produce the same cytogenetic effect observed under combined exposure conditions. Using a linear-quadratic dose–response model typical of low-LET gamma ray calibration curve produced by Abe et al. [36]: Y = 0.0013 + 0.0067D + 0.0313D^2^ (r = 0.9985) we calculated that a dicentric yield of 0.113 corresponds to an effective gamma ray dose of approximately 1.79 Gy, confirming the contribution of UVB in amplifying DNA damage beyond that induced by gamma rays alone. The dicentric yields observed after combined exposure corresponded to gamma equivalent doses of approximately 1.26 Gy, 1.79 Gy, and 1.78 Gy for donors 1, 2, and 3, respectively. Propagating the 95% confidence intervals on dicentric yield (Wilson, N = 500 metaphases) through the calibration curve resulted in gamma equivalent dose intervals of 1.09–1.50 Gy for donor 1, and 1.61–1.99 Gy and 1.60–1.98 Gy for donors 2 and 3, respectively.

Applying BEIR VII risk model for low-LET ionizing radiation [35] to the equivalent dose of 1.79 Gy suggests an excess lifetime cancer risk of around 8.9% (8.1–10%). Similarly, for the 1st donor, a dicentric frequency of 0.059 was observed following co-exposure to 1 Gy gamma rays and 100 J/m^2^ UVB. Using the same linear-quadratic dose–response model [36] we estimated that this aberration yield corresponds to an effective gamma ray dose of approximately 1.26 Gy. The estimated excess cancer risk for this donor under co-exposure conditions is approximately 6.3% (5.4–7.5%). This value is still higher than the baseline risk associated with 1 Gy exposure alone and further supports the synergistic genotoxic potential of combined gamma and UVB radiation. The lower equivalent dose reflects inter-individual variability in cytogenetic response and reinforces the importance of donor-specific biodosimetry in mixed radiation exposure scenarios. For the 3rd donor, results clearly demonstrate a synergistic genotoxic effect from co-exposure to 1 Gy gamma rays and 100 J/m^2^ UVB, as according to Abe et al.’s linear-quadratic dose–response model, the measured dicentric yield of about 0.112 dicentrics/cell equates to an effective gamma-ray dosage of roughly 1.78 Gy and using the BEIR VII cancer risk model, this dose corresponds to an estimated extra lifetime cancer risk of 8.8% (8–9.9%). It is important to highlight that gamma equivalent doses derived from dicentric yields should be interpreted as biodosimetric heuristics rather than direct predictors of individual cancer risk. These values serve to place the biological impact of the co-exposure to gamma rays and UVB within the framework of established gamma-ray dose–response curves, but they do not account for whole-body dose distribution, repair kinetics, or tissue-specific radiosensitivity. The subsequent conversion to an estimated lifetime cancer risk (5% per Gy) assumes a linear–no-threshold (LNT) relationship, following BEIR VII risk coefficients that were derived for in vivo whole-body gamma rays exposures. Therefore, these estimates should thus be viewed as comparative biological indicators rather than quantitative epidemiological predictions.

The aforementioned findings emphasize the possibility of synergistic interactions between ionizing and non-ionizing radiation amplifying DNA damage and, thus, carcinogenic potential. Beyond cancer, ionizing radiation has been linked to a variety of non-cancer health concerns, particularly when exposure is moderate to high or affects sensitive tissues. Radiation-induced cataracts are a well-documented deterministic consequence, with even modest doses (0.5 Gy) causing opacities in the eye’s lens [37]. Cardiovascular effects have also gained significant attention in recent years; studies have shown that radiation doses exceeding 0.5–1.0 Gy to the heart or major arteries are associated with increased risk of ischemic heart disease and stroke [38,39]. Chronic exposure has also been implicated in promoting atherosclerosis and microvascular damage, possibly through persistent inflammation and endothelial dysfunction [40]. Additionally, high-dose radiation to the brain, especially in children, has been linked to neurocognitive impairments and neuroendocrine dysfunctions [41,42].

The use of cytogenetic assays, such as the dicentric chromosome assay, remains a gold standard for estimating absorbed radiation doses and offers insight into potential long-term health effects, including cancer risk [43,44,45]. The findings of this study reinforce the need to consider combined exposures in radiation protection frameworks, particularly in clinical, occupational, or environmental settings where individuals may be exposed to both ionizing and non-ionizing radiation sources. In addition to increased dicentric chromosome formation, our study revealed a notable presence of acentric fragments in peripheral blood lymphocytes following co-exposure to 1 Gy gamma rays and 100 J/m^2^ UVB. The simultaneous elevation of both aberration types suggests distinct yet complementary damage pathways activated by the combined exposure. The mechanistic differences between dicentrics and fragments become especially relevant in the context of combined gamma rays and UVB exposure [46]. Gamma rays induce DSBs via direct ionization and, indirectly, through ROS production. UVB, although being a non-ionizing radiation, causes bulky DNA lesions such as cyclobCPDs and 6–4 photoproducts, which primarily stall DNA replication forks and activate the NER pathway [47,48]. When combined, UVB may inhibit accurate DSB repair by either saturating repair machinery or interfering with signaling cascades such as ATM/ATR and p53. This replication stress can convert UVB-induced lesions into DSBs during the S-phase, further compounding the damage initiated by gamma rays. The net result is both increased misrejoining (yielding dicentrics) and accumulated unrepaired breaks (leading to fragments) [49]. Biologically, the presence of both dicentrics and fragments indicates not only enhanced chromosomal instability but also increased cell heterogeneity, which is a known cause of clonal evolution and cancer [50]. While dicentrics are unstable and often removed during cell division, acentric fragments can persist longer and may be incorporated into micronuclei, contributing to chromothripsis-like events and long-term genomic instability [8,51,52]. Consistent with this mechanistic distinction, in donor 4, PBMCs displayed a higher frequency of acentric fragments relative to whole blood, whereas dicentric yields remained comparable. This discrepancy likely reflects transient or cell–cycle–dependent acentric breakage rather than an increase in true misrepair events, since dicentrics are the most stable indicator of misrepaired DNA DSBs. Such divergence between fragments and dicentrics in individual donors has been reported previously and is attributed to the lower structural stability of acentric lesions and their dependence on cell-cycle stage and replication timing rather than on misrepair frequency [48]. This pattern indicates that the elevated fragment frequency reflects transient breakage events rather than increased misrepair, consistent with the stability difference between fragments and dicentrics. Our findings expand the understanding of radiation-induced oxidative stress and inflammation by highlighting how combined ionizing and non-ionizing exposures interact synergistically to enhance redox imbalance, genomic instability, and potential cancer risk.

## 5. Conclusions

Exposing PBMCs to both gamma rays and UVB radiation causes a synergistic increase in DNA damage, as shown by increased dicentric formation and γH2AX signaling. To forecast dicentric yields, an enhanced linear-quadratic model was devised, which included interaction terms that were dependent on dosage time and radiation sequence. The model correctly captured observed patterns across several donors and sample types, demonstrating its usefulness in mixed-radiation biodosimetry. These findings emphasize the significance of exposure context and encourage the use of synergy-based modeling in future radiation risk evaluations. Combined gamma and UVB exposure synergistically increase DNA damage, likely through cumulative oxidative stress and redox imbalance. Whole-blood antioxidant capacity partially mitigates this effect, underscoring the importance of systemic antioxidant defenses in radiation-induced inflammation and genomic stability.

## Figures and Tables

**Table 1:** Repair index (RI) for each donor under all exposure conditions, both in whole blood (WB) and isolated PBMCs.

Donors	RI-1 Gy Gamma Rays	RI-1 Gy Gamma Rays + 100 J/m^2^
1st	WB: 0.45	WB: 0.30
	PBMCs: 0.67	PBMCs: 0.11
2nd	WB: 0.66	WB: 0.33
	PBMCs: 0.56	PBMCs: 0.14
3rd	WB: 0.65	WB: 0.27
	PBMCs: 0.65	PBMCs: 0.02

**Table 2:** Observed (obs) and expected (exp) biological effect induced by the combination of gamma and UVB radiation and Bliss excess values (Δ) for each donor at 24 h post-irradiation, both in whole blood (WB) and in isolated PBMCs, calculated using the Bliss Independence Model.

	Whole Blood			Isolated PBMCs		
	1st Donor	2nd Donor	3rd Donor	1st Donor	2nd Donor	3rd Donor
$E_{gamma-UVB}^{obs}$	1	1	1	1	1	1
$E_{gamma-UVB}^{exp}$	0.855	0.569	0.623	0.631	0.662	0.797
Bliss excess (Δ)	0.145	0.431	0.377	0.369	0.338	0.203

## Data Availability

All the data we generated are included in the manuscript figures. All logical requests should be addressed to the corresponding author.

## Supplementary Materials

The following supporting information can be downloaded at https://www.mdpi.com/article/10.3390/antiox14121451/s1. Table S1: Studies reporting both DNA damage endpoints and oxidative stress/antioxidant biomarker changes. Table S2: Values of erythemal weighted irradiances Eff (W/m^2^) in different heights. Table S3: Values of absolute irradiances based on the spectral conversion factor given by the manufacturer. Figure S1: Irradiation setups. Table S4: One-tailed paired *t*-tests results for to test weather combined gamma rays and UVB treatment produces higher γH2AX foci than gamma rays alone. Table S5: One-tailed paired *t*-tests results for to test weather combined gamma rays and UVB treatment produces higher γH2AX foci than UVB alone. Table S6: Mean ± 95% confidence intervals (non-parametric bootstrap, 1000 resamples) for all endpoints. γH2AX values are shown for whole blood and PBMCs at 1 h and 24 h. Dicentric chromosome frequencies are shown for whole blood at 48 h. Bliss excess (Δ) summarizes synergy at 24 h for each matrix. Table S7: Donor-level effect sizes for γH2AX and dicentric endpoints, reported as Δ (Combined−Gamma) and Ratio (Combined/Gamma), demonstrating inter-individual consistency of the mixed-field effect. Table S8: Sensitivity of Bliss excess (Δ) to normalization scheme in whole blood and PBMCs. Figure S2: Dose effect curve in PBMCs of the 1st donor. Table S9: Synergy assessed using the HSA (Highest Single Agent) model. References [53,54,55,56,57] are cited in the Supplementary Materials.
