# Supplementary material for: Synergistic Genotoxic Effects of Gamma Rays and UVB Radiation on Human Blood
Source: Antioxidants (Basel). 2025 Dec 2;14(12):1451. doi: 10.3390/antiox14121451 (PMC12729379; doi:10.3390/antiox14121451)
Supplement: Supplementary file 1 [file antioxidants-14-01451-s001.zip › antioxidants-3958281-supplementary.pdf]

Supplementary data

Synergistic Genotoxic Effects of Gamma Rays and UVB Radiation on Human Blood

Angeliki Gkikoudi [1,2], Athanasia Adamopoulou [1], Despoina Diamadaki [1], Panagiotis Matsades [1], Ioannis Tzakakos [1], Sotiria Triantopoulou [2], Spyridon N. Vasilopoulos [1], Gina Manda [3], Georgia I. Terzoudi [2] and Alexandros G. Georgakilas [1,*]

[1] DNA Damage Laboratory, Physics Department, School of Applied Mathematical and Physical Sciences, National Technical University of Athens (NTUA), Zografou Campus, 15780 Athens, Greece; angelikigkikoudi@mail.ntua.gr (A.G.); ge19143@ntua.gr (A.A.); ge20004@mail.ntua.gr (D.D.); ge19811@mail.ntua.gr (P.M.); ge20031@mail.ntua.gr (I.T.); svasilopoulos@mail.ntua.gr (S.N.V.)
[2] Health Physics, Radiobiology & Cytogenetics Laboratory, Institute of Nuclear & Radiological Sciences & Technology, Energy & Safety, National Centre for Scientific Research "Demokritos", 15341 Agia Paraskevi, Greece; iro@rrp.demokritos.gr (S.T.); gterzoudi@rrp.demokritos.gr (G.I.T.)
[3] Radiobiology Laboratory, "Victor Babeș" National Institute of Pathology, 99-101 Splaiul Independentei, 050096 Bucharest, Romania; gina.manda@ivb.ro
* Correspondence: alexg@mail.ntua.gr; Tel.: +30-210-7724453

S1. Literature benchmarks: studies reporting both DNA damage endpoints and oxidative stress / antioxidant biomarker changes after radiation

Various studies have found to provide a structured link between radiation-induced DNA damage and expected oxidative biomarker changes and can be utilized to interpret our findings (Table S1).

Table S1: Studies reporting both DNA damage endpoints and oxidative stress / antioxidant biomarker changes.

Study	Radiation/ UV Exposure Conditions	DNA Damage/ Persistence Endpoint(s)	Oxidative/ Antioxidant Biomarker(s) Reported	Relevance
[1]	High-LET ionizing radiation (dose- and LET-dependent, chronic exposure)	Persistent γH2AX foci and delayed repair of clustered DNA lesions following high-LET exposure.	8-oxo-dG in tissues and biofluids (1.5–2× baseline increases) and antioxidant enzyme modulation.	Persistent DNA oxidation correlates with long-term γH2AX foci persistence and oxidative imbalance.
[2]	Low-dose ionizing radiation from modern biphasic abdominal–pelvis CT in human subjects (ED ≈ 6–9 mS.	Significant γH2AX foci increase 15% in peripheral lymphocytes	Antioxidant enzymes (SH-Px, SOD, and CAT) activities increased by 10–26 % but no significant changes in	Low-dose diagnostic ionizing radiation induces oxidative stress and γH2AX-detectable DNA damage, supporting an Oxidative stress-mediated indirect damage mechanism

			Plasma F2-isoprostanes and FRAP (total antioxidant capacity).	and the potential for antioxidant-based radioprotection.
[1]	Whole-body exposure of Apc1638N/+ mice to low-dose (10 cGy) high-LET ^{28}Si ions vs. γ-rays, assessed at 60 and 150 days post-irradiation.	Persistent intestinal oxidative DNA damage (8-oxo-dG) at 60–150 days, accompanied by increased crypt cell proliferation (Cyclin D1) and metaplasia (GUCY2C), leading to higher tumor burden.	Serum and tissue 8-oxo-dG levels remained elevated (~2×) over time.	Persistent intestinal 8-oxo-dG levels correlated with increased crypt cell proliferation, metaplasia, and higher tumor incidence and invasiveness, linking sustained oxidative DNA damage to late tissue and tumor endpoints after high-LET exposure.

Based on the aforementioned studies, observed DNA damage profiles correspond to a moderate-to-high oxidative stress and inflammatory state and therefore the benchmarking allows inference of likely oxidative biomarker responses even if those markers weren't directly measured in the study.

S2. Donor Characteristics

Peripheral blood was obtained from six apparently healthy adult volunteers: two females aged 24 years, two males aged 40 years, and two males aged 24 years. All donors were non-smokers and reported no symptoms of acute or chronic inflammation or infection within the month preceding blood collection. None had a personal history of autoimmune disease, cancer, or other chronic illnesses, and none were under medication known to affect immune or hematopoietic function. Written informed consent was obtained from all participants in accordance with the approved ethics protocol. The study was conducted in accordance with the Declaration of Greece and approved by the NCSR 'Demokritos' bioethics committee (21/12/2023-17 (Date: 21/02/2023)).

S3. Irradiation setups

Whole blood samples and isolated lymphocytes were exposed to gamma rays and UVB radiation as single stressors, and to their combination. Irradiation setups are shown in Figure S1. The UVB emission spectrum of the lamp was independently validated by the Greek Atomic Energy Commission (GAEC). The Commission confirmed that the measured spectral distribution and intensity of the lamp complied with the manufacturer's specifications and met the experimental requirements for UVB irradiation.

Uniformity of UVB lamb

A narrowband UVB lamp (nominal emission peak ~302 nm) was characterized for use in our experiments. Measurements were performed with a Gigahertz-Optik Hazard Lightmeter X13 equipped with the HD-45-HUV erythema-weighted detector. The lamp was allowed to warm up for 10 min until output stabilized. Petri dishes were placed on a detachable base that allowed five discrete heights (H1–H5) beneath the lamp. Two lateral positions (Position A, left; Position B) were defined for reproducibility (Table S2).

Table S2. Values of erythemal weighted irradiances Eff (W/m^2) in different heights.

**Height**	**Eff (Position A) [W/m^2]**	**Eff (Position B) [W/m^2]**	**Mean[W/m^2]**
H1 (30 cm)	5.6	5.38	5.49
H2	7.5	7.2	7.35
H3	10.57	10.15	10.36
H4 (12.7 cm)	15.5	14.8	15.15
H5	23.4	22.2	22.8

To assess lateral homogeneity of the UVB field, effective irradiance was measured at two lateral positions (A and B) for each vertical height. A uniformity index was calculated for every height as: $U = \frac{E_{min}}{E_{mean}}$ [3.1], where Emin is the lower of the two lateral measurements and Emean their average. Across all distances the uniformity index ranged from 0.94 to 0.96, indicating that the minimum irradiance at any point on the exposure area exceeded 94 % of the lateral mean. This demonstrates excellent lateral uniformity of the UV field over the Petri dish area, supporting reproducible and spatially homogeneous dosing.

Effective-to-Absolute Conversion based Manufacturer's input

The manufacturer specifies 2170 $\mu W/cm^2$ (21.7 W/ m^2) at 5 in (≈12.7 cm) and 730 μW /cm^2 (7.3 W/m^2) at 12 in (≈30 cm) for a typical 302 nm lamp. Comparison of our erythemal measurement at ~12.7 cm (H4, 15.5 W/m^2) with the manufacturer's 21.7 W/m^2 absolute irradiance value (Eabs) gives an approximate spectral conversion factor: $k = \frac{E_{eff}}{E_{abs}} = 0.71$ [3.2] and assuming similar spectral shape at other heights: $Eabs = \frac{E_{eff}}{0.71}$ [3.3] (Table S3).

Table S3. Values of absolute irradiances based on the spectral conversion factor given by the manufacturer.

**Height**	**Eabs [W/m^2]**	**Time for Eabs=100 J/m^2**
H1 (30 cm)	7.9	12.7
H4 (12.7 cm)	21.7	4.6

Spectral Computation of Absolute Irradiance

To obtain absolute irradiance (Eabs) directly from a lamp's spectral curve the lamp's spectral irradiance was measured Eλ (W m^{-2} nm^{-1}).with (Figure S1c) and integrated over the UV range of interest: $Eabs = \int E_\lambda \, d\lambda$ [3.4]. Following, the erythemal weighted

irradiance for validation was computed: $Eabs = \int E_\lambda\, s(\lambda)\, d\lambda$[3.5]. where s(λ) is the CIE erythema action. Finally, the conversion factor was derived: $k = \frac{E_{eff}}{E_{abs}}$[3.6]. Numerical trapezoidal integration gave: Eabs(280 – 400 nm)=35.76 W/m^2. With the paired erythemally weighted meter reading at that geometry (Height 5, Position A), Eeff=23.4 W/m^2 the spectrum-based conversion factor was computed: k≡0.65. The spectrum-derived value gives an absolute irradiance about 9 % higher (and a correspondingly shorter exposure time) than the 0.71 factor inferred from the manufacturer's typical figures.

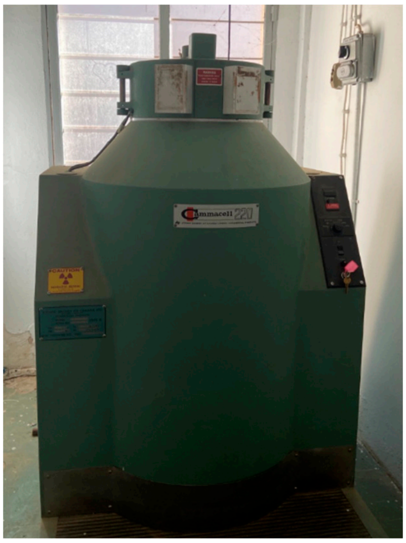

(**a**)

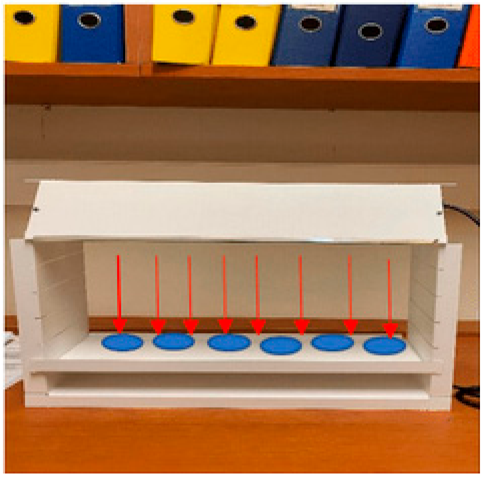

(**b**)

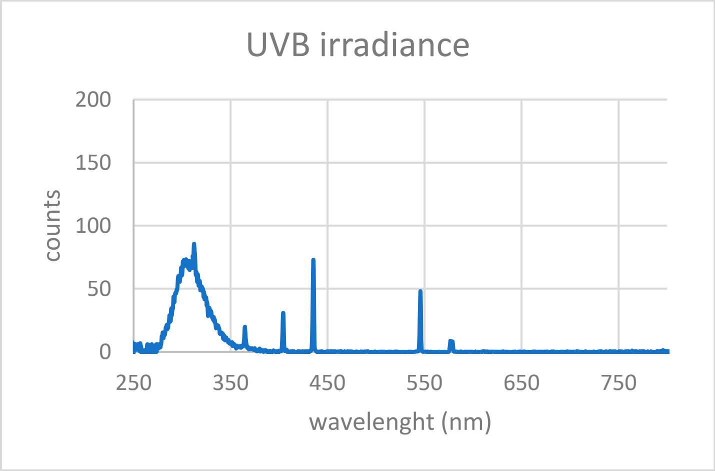

(**c**)

Figure S1. Irradiation setups. (a) Co^{60} source in NCSR "Demokritos; (b) UV Bench Lamp Model XX-15M and (c) UVB emission spectrum of the lamp illustrating the wavelength distribution during experiments.

Erythema Dose Calculation

The erythema dose Deff (J/m²) quantifies the biologically weighted UV exposure and is computed directly from the erythemally weighted irradiance Eeff measured by the HD-45-HUV detector. Assuming stable output during the exposure, the dose is: $D_{eff} = E_{eff} \cdot t$ [3.7], where t is the irradiation time in seconds (1 W/m² = 1 J/ (sm²)). For a desired erythema dose, the required exposure time is therefore $t = E_{eff} \cdot D_{eff}$ [3.8]. To illustrate the dose calculation, consider Height 1 (≈30 cm) at Position A, where the erythemally weighted irradiance was measured at E_eff = 5.6 W m⁻². Equation [3.8] for a 13 s exposure delivers: Deff ≈72.8J/m² , where this is roughly 0.73 SED (SED is internationally used unit that expresses UV radiation in terms of its ability to cause erythema (sunburn) and 1 SED = 100 J/m²) [3,4].

S4. Statistical analysis-Bootstrapped Confidence Intervals, Donor-Level Effects, and Synergy Analyses

For each donor comparisons where performed, for WB and PBMCs, at 1 h and 24 h post-exposure. Two technical replicates were available for each condition (df = 1). One-tailed paired t-tests were applied to test the directional hypothesis that the combined gamma rays and UVB treatment produces higher γH2AX foci than gamma rays or UVB alone. Given the small number of biological replicates (n = 3 donors) and the exploratory scope of the study, we did not apply formal multiple-comparison corrections. Instead, non-parametric bootstrapping (1000 iterations) was used to derive 95 % confidence intervals and assess robustness against violations of normality assumptions.

Table S4: Two-tailed unpaired t-tests results for to test weather combined gamma rays and UVB treatment produces higher γH2AX foci than gamma rays alone.

Donor	Matrix	Time (h)	t	df	p
1	WB	1	2.02619246	37	0.33912818
1	WB	24	2.04227246	30	0.03568194
2	WB	1	2.00171748	58	0.46051966
2	WB	24	2.00758377	51	1.2919E-06
3	WB	1	1.99713791	65	0.58145144
3	WB	24	1.99546893	68	1.6059E-08
1	PBMC	1	1.96888862	267	0.98350343
1	PBMC	24	1.97471579	162	1.6792E-18
2	PBMC	1	1.97993041	120	0.42220599
2	PBMC	24	1.99210215	75	4.688E-13
3	PBMC	1	1.9869787	89	0.85424564
3	PBMC	24	1.9925435	74	2.4323E-25

Table S5: Two-tailed unpaired t-tests results for to test weather combined gamma rays and UVB treatment produces higher γH2AX foci than UVB alone.

Donor	Matrix	Time (h)	t	df	p
1	WB	1	2.03224451	34	2.3891E-08
1	WB	24	2.028094	36	0.13615347
2	WB	1	2.01063476	48	3.8321E-09
2	WB	24	1.99772965	64	0.02714512
3	WB	1	2.00324072	56	5.8262E-09
3	WB	24	1.99210215	75	0.01241559
1	PBMC	1	1.96878902	270	8.0839E-30
1	PBMC	24	1.97149039	207	7.2262E-06
2	PBMC	1	1.98760828	87	5.0466E-15
2	PBMC	24	1.97756078	136	0.01006417
3	PBMC	1	1.98931856	82	1.821E-15
3	PBMC	24	1.98137181	112	4.137E-09

Bootstrapped confidence intervals confirmed robust increases in γH2AX foci and dicentric yields following irradiation, with the highest responses under co-exposure. Bliss excess was positive across all donors and in both matrices, supporting a reproducible synergistic interaction beyond Bliss additivity (Table S4 and Table S5).

Table S6: Mean ± 95% confidence intervals (non-parametric bootstrap, 1,000 resamples) for all endpoints. γH2AX values are shown for whole blood and PBMCs at 1 h and 24 h. Dicentric chromosome frequencies are shown for whole blood at 48 h. Bliss excess (Δ) summarizes synergy at 24 h for each matrix.

Endpoint	Matrix	Time (h)	Condition	Mean	95% CI lower	95% CI upper
γH2AX	Whole blood	1	Control	1.945	1.79	2.23
γH2AX	Whole blood	1	Gamma	6	5.42	6.29
γH2AX	Whole blood	1	UVB	2.677	2.52	2.79
γH2AX	Whole blood	1	Combined	6.502	6.065	6.89
γH2AX	Whole blood	24	Control	1.945	1.79	2.23
γH2AX	Whole blood	24	Gamma	2.515	1.98	3.43
γH2AX	Whole blood	24	UVB	3.55	3.37	3.85
γH2AX	Whole blood	24	Combined	4.55	4.405	4.795
γH2AX	PBMC	1	Control	1.402	1.065	1.65
γH2AX	PBMC	1	Gamma	6.148	5.9	6.31
γH2AX	PBMC	1	UVB	2.962	2.425	3.41
γH2AX	PBMC	1	Combined	6.3	6.26	6.35
γH2AX	PBMC	24	Control	1.402	1.065	1.65
γH2AX	PBMC	24	Gamma	2.235	2.035	2.44

γH2AX	PBMC	24	UVB	3.962	3.48	4.27
γH2AX	PBMC	24	Combined	5.65	5.2	6.14
Dicentrics	Whole blood	48	Control	0	0	0
Dicentrics	Whole blood	48	Gamma	0.045	0.021	0.059
Dicentrics	Whole blood	48	UVB	0.013	0.003	0.028
Dicentrics	Whole blood	48	Combined	0.096	0.059	0.114
Bliss excess	Whole blood	24	—	0.318	0.145	0.431
Bliss excess	PBMC	24	—	0.303	0.203	0.369

Table S7: Donor-level effect sizes for γH2AX and dicentric endpoints, reported as Δ (Combined − Gamma) and Ratio (Combined / Gamma), demonstrating inter-individual consistency of the mixed-field effect.

**Endpoint**	**Matrix**	**Time (h)**	**Donor**	**Gamma**	**Combined**	**Δ (Combined − Gamma)**	**Ratio (Combined / Gamma)**
γH2AX	Whole blood	1	1	6.29	6.89	0.6	1.095
γH2AX	Whole blood	1	2	6.29	6.55	0.26	1.041
γH2AX	Whole blood	1	3	5.42	6.065	0.645	1.119
γH2AX	Whole blood	24	1	3.43	4.795	1.365	1.398
γH2AX	Whole blood	24	2	2.135	4.405	2.27	2.063
γH2AX	Whole blood	24	3	1.98	4.45	2.47	2.247
γH2AX	PBMC	1	1	6.235	6.26	0.025	1.004
γH2AX	PBMC	1	2	5.9	6.35	0.45	1.076
γH2AX	PBMC	1	3	6.31	6.29	-0.02	0.997
γH2AX	PBMC	24	1	2.035	5.61	3.575	2.757
γH2AX	PBMC	24	2	2.44	5.2	2.76	2.131
γH2AX	PBMC	24	3	2.23	6.14	3.91	2.753
Dicentrics	Whole blood	48	4	0.021	0.059	0.038	2.759
Dicentrics	Whole blood	48	5	0.055	0.113	0.058	2.04
Dicentrics	Whole blood	48	6	0.059	0.114	0.055	1.94

For the sensitivity analysis in Table S8, normalization was applied within each donor's dataset prior to computing Bliss excess (Δ). Specifically, in the *per-donor min–max* and *per-donor z-score* schemes, each donor's raw γH2AX values were first rescaled independently to remove differences in baseline or variability. Bliss excess was then calculated separately for each donor, and the resulting Δ values were averaged across the three donors. The table therefore reports the mean Δ ± 95 % bootstrap confidence interval summarizing inter-donor variation under each normalization approach. For comparison, the *global min–max* normalization used a single 0–1 scaling based on all donors combined. This analysis tests the robustness of the synergy finding to different normalization methods rather than re-evaluating individual donor effects.

Table S8: Sensitivity of Bliss excess (Δ) to normalization scheme in whole blood and PBMCs.

Matrix	Normalization	Mean Δ	95% CI lower	95% CI upper
Whole blood (24 h)	Absolute (global min–max)	0.248	0.143	0.329
Whole blood (24 h)	Min–max per donor	0.317	0.145	0.43
Whole blood (24 h)	Z-score per donor (Φ)	0.16	0.077	0.213
PBMC (24 h)	Absolute (global min–max)	0.235	0.103	0.32
PBMC (24 h)	Min–max per donor	0.313	0.233	0.369
PBMC (24 h)	Z-score per donor (Φ)	0.177	0.122	0.222

S5. Dose–effect curve in isolated donor lymphocytes

To evaluate the relationship between absorbed gamma radiation dose and DNA double-strand break (DSBs) formation, peripheral blood mononuclear cells (PBMCs) isolated from the first donor were exposed to increasing doses of ^{60}Co gamma rays. DNA damage was assessed by quantifying γH2AX foci per nucleus. The resulting dose-response relationship is presented below ~ 7.2 DSBs/Gy/cell:

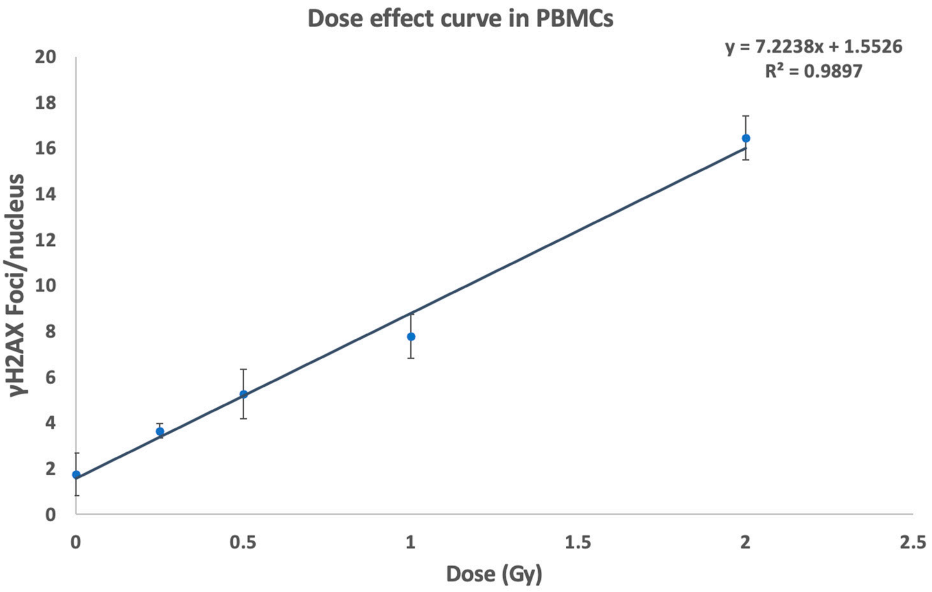

Figure S2. Dose effect curve in PBMCs of the 1st donor. Mean number of γH2AX foci per nucleus as a function of absorbed dose (0–2 Gy). Points represent mean ± SD of two independent experiments; the line indicates linear regression ($y = 7.2238x + 1.5526$, $R^2 = 0.9897$).

S6. HSA (Highest Single Agent)

The HSA (Gaddum's noninteraction) model defines synergy by calculating the CI parameter: $CI = \max(E_A, E_B)/E_A+E_B$ [6.1] (CI<1→ Synergy, CI=0→additivity and CI>1→antagonism) [6]. Donor-level effects (γH2AX foci per cell) were used to compute CI values. Both matrices (whole blood and PBMCs) yielded CI < 1, confirming synergistic co-exposure responses (Table S9).

Table S9: Synergy assessed using the HSA (Highest Single Agent) model.

Matrix	Donor	Eγ	EUVB	Eγ+UVB	max(Eγ,EUVB)	CI = max / Eγ+UVB
Whole blood (24 h)	1	3.43	3.85	4.795	3.85	0.803
Whole blood (24 h)	2	2.135	3.43	4.405	3.43	0.779
Whole blood (24 h)	3	1.98	3.37	4.45	3.37	0.757
PBMC (24 h)	1	2.035	3.48	5.61	3.48	0.62
PBMC (24 h)	2	2.44	4.135	5.2	4.135	0.795
PBMC (24 h)	3	2.23	4.27	6.14	4.27	0.695

S7. Parameter Selection for the Extended Linear–Quadratic (LQ) Model

Values of the key model parameters (e.g. α, β, γ, δ) used in the extended linear–quadratic formulation: $Ydic=\alpha D+\beta D^2+\gamma D_{UVB}+\delta DD_{UVB}e^{-\Delta t/20}\cdot w_{order}$ [7.1],

were derived by integrating data from authoritative bibliographic sources with the experimental outcomes of the present study. In particular:

- α (linear term) and β (quadratic term) describe dicentric induction by low-LET gamma rays. These terms were extracted from published calibration curves of human peripheral blood lymphocytes [5], which report typical values of $\beta \approx 0.01$ Gy^{-2} and $\alpha \approx 0.05$ Gy^{-1} in the 0–2 Gy dose range.
- γ (UVB term) was introduced to capture any independent contribution of UVB to dicentric formation. This term was limited to a small value (≈ 0.001–0.005 $(J/m^2)^{-1}$) and optimised against our measurements of UVB-only samples, which produced near-background dicentric levels, because UVB cytogenetics literature consistently shows minimal yield at the fluences used (≤ 100 J/m^2).
- δ (synergy coefficient) quantifies the interaction between gamma and UVB. Since there is no direct bibliographic value, this term was calculated by fitting the model to the combined-exposure data for all donors. This was done in order to target the empirically observed approximately twofold increase in dicentrics when compared to 1 Gy alone. Consistent across donors, the best fit was $\delta \approx 0.007$ $(Gy \cdot J/m^2)^{-1}$.
- w_{order} reflects sequence dependence of exposures, following earlier radiobiology studies of combined UV and X/gamma irradiation [6]. We used $w_{order} = 0.1$ as a default when gamma radiation came before UVB and adjusted this factor (0.2-1) when other sequences were tested.
- Δt was set to the measured 15–20 min delay between gamma and UVB irradiations, consistent with the temporal repair-decay.
- ε represents biological and technical variance and we held this variable constant at 0.01.

References

1. Kumar, K.; Kumar, S.; Angdisen, J.; Datta, K.; Fornace, A.J.; Suman, S. Radiation Quality-Dependent Progressive Increase in Oxidative DNA Damage and Intestinal Tumorigenesis in Apc1638N/+ Mice. *Current Oncology* **2025**, *32*, 382.
2. Ramos, B.; Gómez-Cayupán, J.; Aranis, I.; García Tapia, E.; Coghlan, C.; Ulloa, M.-J.; Gelerstein Claro, S.; Urbina, K.; Espinoza, G.; De Grazia, J.; et al. Oxidative Stress-Mediated DNA Damage Induced by Ionizing Radiation in Modern Computed Tomography: Evidence for Antioxidant-Based Radioprotective Strategies. *Antioxidants* **2025**, *14*, 1085.
3. Guidelines on limits of exposure to ultraviolet radiation of wavelengths between 180 nm and 400 nm (incoherent optical radiation). *Health Phys* **2004**, *87*, 171-186, doi:10.1097/00004032-200408000-00006.
4. Sliney, D.H. Radiometric quantities and units used in photobiology and photochemistry: recommendations of the Commission Internationale de L'Eclairage (International Commission on Illumination). *Photochem Photobiol* **2007**, *83*, 425-432, doi:10.1562/2006-11-14-ra-1081.
5. Duarte, D.; Vale, N. Evaluation of synergism in drug combinations and reference models for future orientations in oncology. *Current Research in Pharmacology and Drug Discovery* **2022**, *3*, 100110, doi:https://doi.org/10.1016/j.crphar.2022.100110.
6. Abe, Y.; Yoshida, M.A.; Fujioka, K.; Kurosu, Y.; Ujiie, R.; Yanagi, A.; Tsuyama, N.; Miura, T.; Inaba, T.; Kamiya, K.; et al. Dose–response curves for analyzing of dicentric chromosomes and chromosome translocations following doses of 1000 mGy or less, based on irradiated peripheral blood samples from five healthy individuals. *Journal of Radiation Research* **2017**, *59*, 35-42, doi:10.1093/jrr/rrx052.
7. Holmberg, M.; Strausmanis, R. The repair of chromosome aberrations in human lymphocytes after combined irradiation with UV-radiation (254 nm) and X-rays. *Mutat Res* **1983**, *120*, 45-50, doi:10.1016/0165-7992(83)90072-6.
